# miR-205 is involved in metastatic potential of 21T series, a breast cancer progression model

**DOI:** 10.1186/1753-6561-7-S2-P44

**Published:** 2013-04-04

**Authors:** Luiza Stankevicins, Ana Barat, Yegor Vassetzky, Claudia Vitória de Moura Gallo

**Affiliations:** 1 Departamento de Genética, Universidade do Estado do Rio de Janeiro, Instituto de Biologia Roberto Alcantara Gomes, Rio de Janeiro, Brazil; 2 Université Paris-Sud 11 CNRS UMR 8126 «Signalisation, noyaux et innovations en cancérologie», Institut de Cancérologie Gustave-Roussy, Université Paris-Sud 11, Villejuif, France

## Background

microRNA (miRNA) is a class of non-coding RNAs, which regulate gene expression at the post-transcriptional level. Many miRNAs have been implicated in several human cancers, as these regulatory molecules play important roles in key cellular processes, including cell proliferation, differentiation and response to DNA damage.

## Methods

To gain insights into the mechanisms involved in breast cancer initiation and progression we conducted a miRNA global expression on 21T series. These cell lines represent an *in vitro* model of breast cancer progression comprising immortalized cell lines derived from the same patient diagnosed with Her-2 overexpressing ductal carcinoma. The set include a normal epithelia (16N), primary *in situ* ductal carcinoma (21PT and 21NT) and cells derived from pleural effusion of lung metastasis (21MT-1 and 21MT-2). To confirm microarray results, the expression of the most significantly altered miRNAs were checked by qPCR. Matrigel invasion assay was done to evaluate the migration capacity of 21Tcells and Her-2, ZEB-1 and e-cadherin protein levels were achieved by western blot.

## Results

Analysis of 21T series revealed a significant downregulation of miR-205 together with an enrichment of its predicted target, the pro-metastatic factor *ZEB-1* and the consequent reduction of e-cadherin levels in the invasive 21MT cells.

## Conclusions

These molecular alterations, in special the downregulation of miR-205 in cancer cells, can participate on modulation of epithelial to mesenchymal transition and increase the metastatic potential on breast cancer.

## Financial support

Faperj, CAPES, CNPq and Université Paris XI.

